# Marked Rise in the Prevalence of Asymptomatic *Plasmodium falciparum* Infection in Rural Gabon

**DOI:** 10.1371/journal.pone.0153899

**Published:** 2016-05-26

**Authors:** Irène Pegha Moukandja, Jean Claude Biteghe Bi Essone, Issaka Sagara, Roland Fabrice Kassa Kassa, Julien Ondzaga, Jean-Bernard Lékana Douki, Marielle Bouyou Akotet, Dieudonne Nkoghe Mba, Fousseyni S. Touré Ndouo

**Affiliations:** 1 Centre International de Recherches Médicales de Franceville (CIRMF) B.P. 769 Franceville, Gabon; 2 Ecole Doctorale Régionale (EDR) en Infectiologie Tropicale, BP: 876 Franceville, Gabon; 3 Département de Parasitologie-Mycologie Médecine Tropicale, Faculté de Médecine, Université des Sciences de la Santé, B.P. 4009 Libreville, Gabon; 4 Département d'Epidémiologie et des Affections Parasitaires, MRTC, Faculté de Médecine et d'Odontostomatologie, Université de Bamako, BP 1805 Bamako, Mali; Université Pierre et Marie Curie, FRANCE

## Abstract

Control strategies implemented a decade ago led to a marked reduction in the prevalence of malaria in many countries. In Dienga, southeastern Gabon, the prevalence of microscopic *P*. *falciparum* infection was 7% in 2003, close to the pre-elimination threshold of 5%. The aim of this work was to determine the prevalence of *P*. *falciparum* infection in the same community a decade later. A cohort of 370 individuals aged from 3 to 85 years living in Dienga was investigated for *P*. *falciparum* infection; during six passages (P) in 15-month period. Demographic data were collected, along with behaviors and attitudes towards malaria. *Plasmodium* infection was diagnosed by microscopy (ME), followed by PCR to detect submicroscopic infection. The prevalence of *P*. *falciparum* infection in P1, P2, P3, P4, P5 and P6 was respectively 43.5% (25.1% ME+, 18.4% PCR+); 40.9% (27.0% ME+, 13.9% PCR+), 52.7% (26.1% ME+, 26.6% PCR+); 34.1% (14.1% ME+, 20% PCR+), 57.7% (25.4.% ME+, 32.3% PCR+); and 46.2% (21.4% ME+, 24.8% PCR+) with an overall average of 45.9% (95%CI [37.0–54.7], 23.2% ME+ and 22.7% PCR+). P4 and P5 prevalences were statically different throughout the six passages. Microscopic prevalence was significantly higher than that observed ten years ago (23% [n = 370] vs 7% [n = 323], p < 0.001). Asymptomatic infections were the most frequent (96%). Gametocytes were detected in levels ranging from 5.9% to 13.9%. Insecticide-treated nets, indoor residual insecticides, and self-medication were used by respectively 33.2% (95%CI [29.0–37.4]), 17.7% (95%CI [15.5–19.9]) and 12.1% (95%CI [10.6–13.6]) of the study population. A near-threefold increase in *P*. *falciparum* infection has been observed in a rural area of southeastern Gabon during a 10-year period. Most infections were asymptomatic, but these subjects likely represent a parasite reservoir. These findings call for urgent reinforcement of preventive measures.

## Introduction

In 2013 there were 135–287 million new cases of malaria and 473 000–789 000 deaths [1]. Among the five species which can infect humans, *Plasmodium falciparum* and *P*. *vivax* are responsible for most morbidity and *P*. *falciparum* for most deaths, mainly among children under the age of 5 years living in sub-Saharan Africa.

Clinical malaria is defined by intermittent fever, malaise, shaking, chills, arthralgia, myalgia, vomiting and other symptoms, together with malaria parasites in blood [2]. Asymptomatic infection is the state diagnosed by the presence of the pathogen without the corresponding disease symptoms. Asymptomatic malaria can be diagnosed by blood smears showing *Plasmodium* on microscopic examination (ME+) and/or rapid diagnostic tests (RTD) and by PCR detecting sub microscopically infected individuals (SMI) who are negative by ME [3,4]. People living in malaria-endemic countries eventually develop immunity, enabling them to maintain a low parasite burden, below the pyrogenic threshold. Microscopic examination fails to detect as many as half of *Plasmodium* carriers [5]. The existence of these asymptomatically infected individuals could undermine malaria control strategies. Submicroscopic infection can also be associated with severe malaria [6,7]. Chronic untreated *Plasmodium* infection can negatively affect children’s health and education [8,9,10,11]. A fraction of asexual merozoites released from infected red blood cells produce sexual gametocytes that can infect mosquitoes [12] and thereby maintain human transmission [13,14,15,16,17].

More than decade ago, the international community and national partners deployed global efforts to reduce malarial morbidity and mortality. In nearly all endemic countries, including Gabon, the prevalence of *P*. *falciparum* infection declined, likely due mainly to the use of ACT and insecticide-treated bed nets [18,19,20]. Unfortunately, the prevalence of malaria is tending to increase again, including in Gabon [21,22].

The reservoir of asymptomatically infected individuals will need to be dealt with if malaria is to be eradicated. Epidemiological surveillance based on molecular tools can help to plan and monitor malaria control programs. The aim of this study was to assess the prevalence of *P*. *falciparum* infection in Dienga, a rural community in southeastern Gabon.

## Materials and Methods

### Study area

The study was conducted in southeast Gabon, in a rural community named Dienga (Ogooué-Lolo province). Dienga is situated near the Congo border and has around 2500 inhabitants. An equatorial climate prevails and is characterized by two rainy seasons (March-May and October-December) and two dry seasons (June-September and January-February). Malaria is highly endemic and *P*. *falciparum* (80%) is predominantly transmitted by *A*. *gambiae*. The entomological infection rate is equivalent to about 100 infective bites per human per year. Malaria in Dienga exhibits seasonal peaks of transmission coinciding with the rains, from February to June and September to December [23].

### Ethical approval

The study was submitted in February 2013 to the “Comité National d’Ethique” (CNE) of Gabon together with a 2-pages questionnaire with which written consent and clinical status were obtained. A month later the study was finally approved and registered under PROT N°0018/2013/SG/CNE with the accord of the Public Health Minister, the Regional Health Director of Ogooué-Lolo province, and Dienga Traditional Chiefs. A meeting was held with the population to explain the study goals and likely benefits.

### Study population

This longitudinal study was conducted from April 2013 to June 2014 during six passages. A calculated sample size of 323 was adjusted to 370, using the following formula N = t^2^*p (1-P)/m^2^, where t is the 95% confidence interval; p is alpha error which corresponds to 1.96 in the normal standard distribution, P is the recently estimated prevalence of *P*. *falciparum* malaria in Dienga (30%) and m is a 5% margin of error. The study population was composed of volunteers living permanently in Dienga; primary school children (≥3 years) and adults. A written informed consent was signed by all participants or their legal tutor before their enrolment.

### Blood collection

Study participants were examined physically, and their axillary temperature and history of fever (≥37.5°C) in the previous 24 hours were recorded on a structured questionnaire. During the following passages: P1 (April 2013), P5 (March 2014) and P6 (June 2014), blood was collected by venipuncture in 5-ml EDTA tubes for thick-film preparation and PCR diagnosis. During P2 (June 2013), P3 (July 2013) and P4 (October 2013), blood samples were collected by finger prick for thick-film preparation and blotted on filter paper for PCR. During each passage the entire population of Dienga was invited to see a CIRMF physician, and any necessary laboratory tests and medications were provided free of charge.

#### DNA extraction

*P*. *falciparum* DNA was extracted from blots by the Chelex method [24]. Briefly, 150 μl of PBS was added to a 1.5-ml microcentrifuge tube containing three or four pieces of filter paper, then vortexed and incubated for 15 min at room temperature. The PBS was removed while keeping the filter paper at the bottom of the tube. Then 200 μl of 5% Chelex-100 was added and heated at 100°C for 15 min. The tube was vortexed every 2 minutes during the heating phase, with the Chelex pressed against the filter paper. The mixture was then centrifuged at 8000 *g* for 1 min and the final supernatant containing DNA was stored at −20°C until use.

DNA was extracted from whole blood by using the DNeasy Blood & Tissue kit according to the manufacturer's procedure (QIAGEN, Hilden, Germany).

### Malaria diagnosis

#### Microscopy

Blood films were prepared as described by Planche *et al*. 2001 [25]. Slides were stained with 10% Giemsa solution for 15 min and examined under a microscope. Samples were considered *Plasmodium-*positive if at least one parasite was seen in a x100 oil-immersion field of a thick blood film (ME+) or when PCR, applied only to ME- samples, was positive (SMI). All PP+ individuals (ME+ and SMI = PCR+) received artemether-lumefantrine according to the national guideline.

#### Molecular diagnosis

PCR targeting Subtelomeric Variable Open Reading frame (STEVOR) genes was used in this study. Primary amplification was performed in a total reaction mixture of 25 μl containing 2.5 μl of x10 reaction buffer, 10 μM each dNTP (dATP, dGTP, dTTP and dCTP), 5 U/μl *Taq* DNA polymerase, 2.5 pM each primer (P5, P18, P19 and P20) and 5.0 μl of DNA extract. The PCR program consisted of denaturation at 93°C for 3 min, 93°C at 50 s, 50 s at 50°C and 50 s at 72°C followed by 25 cycles and a final extension step of 6 min at 72°C. Two microliters of the first-round PCR product was used for nested amplification in a reaction mixture of 22 μl containing 2.5 μl of ×10 reaction buffer, 10 μM each dNTP (dATP, dGTP, dTTP, and dCTP), 5 U/μl of *Taq* DNA polymerase, and 2.5 pM each primer (P17 and P24). The PCR program consisted of denaturation at 93°C for 3 min, 30 s at 93°C, 50 s at 50°C and 50 s at 72°C followed by 40 cycles and a final extension step of 3 min at 72°C. The primer sequences used for STEVOR PCR as previously described [26] are presented in (Table 1).

**Table 1:** Nucleotide primers used in STEVOR gene amplification.

Primer Name	Nucleotide sequence
P5	5'-GGG AAT TCT TTA TTT GAT GAA GAT G-3'
P18	5'-TTT CA(C/T) CAC CAA ACA TTT CTT-3'
P19	5'-AAT CCA CAT TAT CAC AAT GA-3'
P20	5'-CCG ATT TTA ACA TAA TAT GA-3'
P17	5'-ACA TTA TCA TAA TGA (C/T) CC AGA ACT-3'
P24	5'-GTT TGC AAT AAT TCT TTT TCT AGC-3'

PCR products were subjected to electrophoresis on 1% agarose gels and visualized by transillumination with ultraviolet light after staining with EZvision three DNA dye & buffer, 6X (cat. N313, Interchim, France). Fragment sizes were measured relative to a standard size marker (100 bp DNA ladder). All reactions were run in parallel with DNA from negative controls (unexposed European samples) and positive controls (40,000 *P*. *falciparum* parasites/μl on thick blood smears).

### Survey of attitudes and behaviors

An oral survey was conducted in March 2014 using a questionnaire written in French and focusing on the use of insecticide-treated bed nets (ITNs), indoor residual sprays (IRS) and self-medication. Individuals were asked if they had a bed net, if they had used it the night before the interview, and if the net was treated with an insecticide. Similar questions on IRS and self-medication were posed. The parents or guardians answered for children younger than 16 years, during home by home visit. The answers to the questionnaire were stored in electronic form. Ten percent of oral responses of ITNs and IRS were checked.

### Data processing and analysis

All data were recorded in an electronic database. Epi Info 5.4 software was used for conventional descriptive analysis that consisted of calculating proportions (for qualitative data), or mean ± standard deviation, median and range (for quantitative data). Comparative analyses were carried out using the Chi-square test for frequencies (proportions) and Student's t test for means. Univariate analysis was used to describe malaria infection, the dependent variable using Info 5.4 software. Multivariate analysis was performed using STATA 14.0 (Texas 77845 USA, 4905 Lakeway Drive College Station). Then, independent variables such as age, sex, use of ITNs, IRS and self-medication were considered in multivariate analysis adjusting for confounders. Significance was assumed at p<0.05.

## Results

### Study site

The study was conducted in southeast Gabon, in a rural community named Dienga (Ogooué-Lolo province). Dienga is situated near the Congo border and has around 2500 inhabitants. An equatorial climate prevails and is characterized by two rainy seasons (March-May and October-December) and two dry seasons (June-September and January-February). Malaria is highly endemic and *P*. *falciparum* (80%) is predominantly transmitted by *A*. *gambiae*. The entomological infection rate is equivalent to about 100 infective bites per human per year. Malaria in Dienga exhibits seasonal peaks of transmission coinciding with the rains, from February to June and September to December [23].

### Sociodemographic characteristics

A total of 370 participants (schoolchildren and adult volunteers) were included in this longitudinal study from April 2013 to June 2014. The prevalence of *P*. *falciparum* infection was determined during six passages (P) (April 2013 (P1), June 2013 (P2), July 2013 (P3), October 2013 (P4), March 2014 (P5) and June 2014 (P6)). It was an open cohort and the participants were not necessarily the same number at each passage. The individuals were divided into five age groups: [3–6 y], [7–10 y], [11–14 y], [15–18 y] and [19 y≥]. There were slightly more males than females in July 2013 (53.7%) and June 2014 (54.5%) (Table 2).

**Table 2:** Baseline Characteristics of the study subjects.

	Apr-13	Jun-13	Jul-13	Oct-13	Mar-14	Jun-14
**Baseline characteristic**						
**N**	370	267	218	255	232	145
**Means age: yrs (± SD)**	24.34 (22.65)	24.79 (22.61)	21.79 (19.32)	21.88 (20.79)	21.87 (20.77)	17.50 (17.15)
**Males (%)**	49.70	50.60	49.10	49.40	49.60	54.50
**Females (%)**	50.30	49.40	50.90	50.60	50.40	45.50
**Distribution by age (%)**						
**3–6 yrs.**	8.60	8.60	9.21	10.20	9.90	8.30
**7–10 yrs.**	27.00	26.60	27.20	28.20	29.30	37.20
**11–14 yrs.**	18.10	16.90	18.47	20.00	20.70	26.20
**15–18 yrs.**	9.50	9.70	9.20	9.80	8.60	8.30
**19 + yrs.**	36.80	38.30	35.92	31.80	31.50	20.00

### Prevalence of *P*. *falciparum* infection

**Fig 1:**
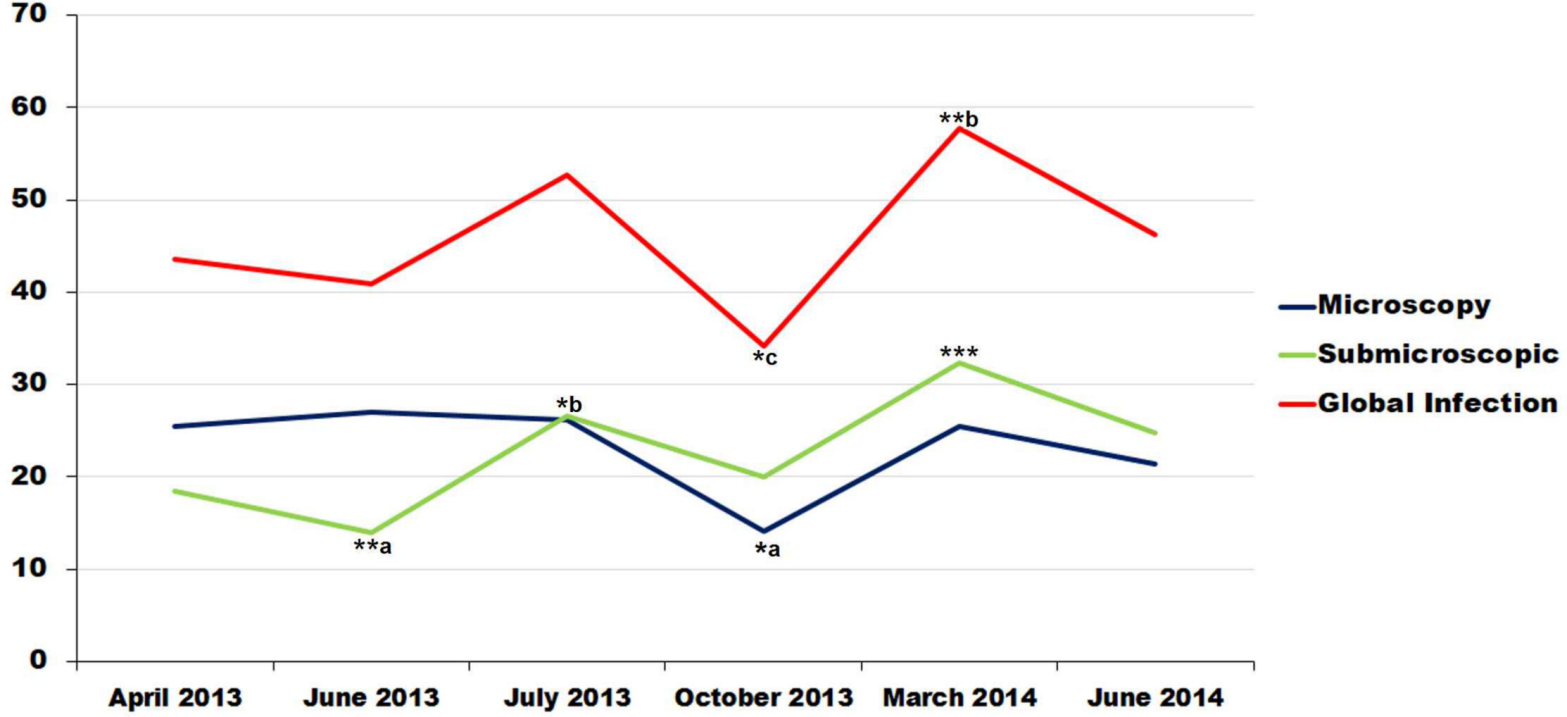
Global prevalence of malaria infection in Dienga. *^a^: *Significant difference between the prevalences of October 2013 and those of other sampling periods except June 2014*, *in microscopic infection*. ** ^**a**^: *Significant difference between the prevalences of June 2013 and those of other sampling periods*, *in submicroscopic infection*. *^b^: *Significant difference between the prevalences of July 2013 and that of June 2013*, *in submicroscopic infection*. ***: *Significant difference between the prevalence of March 2014 and those of other sampling periods except July2013 and June 2014*, *in submicroscopic infection*. *^c^: *Significant difference between the prevalence of October 2013 and that of March 2014*, *in Global infection*. **^b^: *Significant difference between the prevalence of March 2014*, *April and June 2013 in Global infection*.

The frequency of *P*. *falciparum* infection was 161/370 (43.5%) in April 2013 (P1), comprising 93 ME + (25.1%) and 68 (18.4%) PCR+ ME—participants. The corresponding data were 109/267 (40.9%), 72 ME + (27%) and 37 PCR+ (13.9%) in June 2013 (P2); 115/218 (52.7%), 57 (26.1%) ME + and PCR+ (26.6%) in July 2013; 87/255 (37.7%), 36 ME + (14.1%) and 51 PCR+ (20%) in October 2013; 134/232 (57.7%), 59 ME + (25.4%) and PCR+ (32.3%) in March 2014 (P5); and 67/145 (46.6%), 31 ME + (21.4%) and PCR+ (24.8%) in June 2014 (P6). The microscopic prevalence was significantly higher during the first three passages (P1, P2 and P3) than in P4 (P = 0.007). There was also a significant difference between the prevalence of PCR positivity between June 2013 (P2) and July 2013 and March 2014 (p<0.005). No difference in the overall prevalence of *falciparum* infection (ME + plus SMI) was observed across the six passages (p = 0.13) (Fig 1 and Table 3).

**Table 3:** Global *P*. *falciparum* prevalences in Dienga.

Sampling time	n	Microscopy % (IC95%)	Submicroscopic % (IC95%)	Global Infection % (IC95%)	P value
**April 2013**	370	25.4 (20.9–29.9)	18.4 (14.7–22.7)	43.5 (38.4–48.7)	<0.001
**June 2013**	267	27.0 (21.7–32.7)	13.9 (9.9–18.6)**^f^	40.9 (34.9–47.0)	
**July 2013**	218	26.1 (20.4–32.5)	26.6 (20.9–33.0)*^s^	52.7 (45.9–59.5)	
**October 2013**	255	14.1 (10.1–19.0) *^f^	20.0 (15.3–25.4)	34.1 (28.3–40.3) *^t^	
**March 2014**	232	25.4 (20.0–31.5)	32.3 (26.4–38.8)***	57.7 (51.1–64.2) **^s^	
**June 2014**	145	21.4 (15.0–29.0)	24.8 (18.0–32.6)	46.2 (37.9–54.7)	
**Mean prevalence**		23.4 (18.1–28.3)	22.0 (15.8–29.6)	45.4 (37.0–54.7)	

*^f^: Significant difference between the prevalences of October 2013 and those of other sampling periods except June 2014, in microscopic infection.

** ^**f**^: Significant difference between the prevalences of June 2013 and those of other sampling periods, in submicroscopic infection.

*^S^: Significant difference between the prevalences of July 2013 and that of June 2013, in submicroscopic infection.

***: Significant difference between the prevalence of March 2014 and those of other sampling periods except July2013 and June 2014, in submicroscopic infection.

*^t^: Significant difference between the prevalence of October 2013 and that of March 2014, in the Global infection

**^S^: Significant difference between the prevalence of March 2014, April and June 2013 in Global infection.

**Fig 2:**
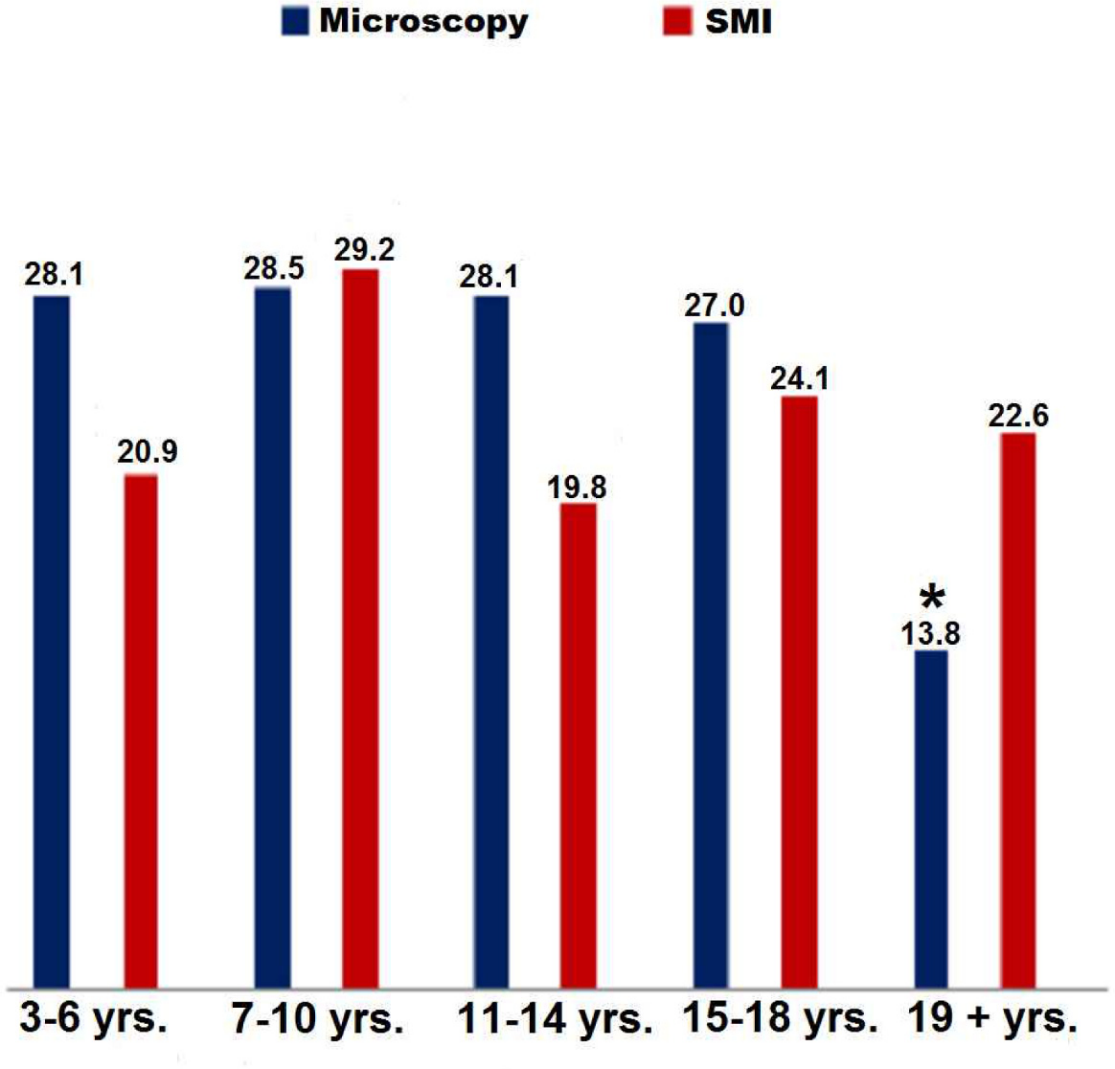
Mean prevalence according to the age groups. * Significant difference between the prevalence of individuals higher than 18years and other groups.

The overall prevalence *falciparum* infection did not differ across the age groups or between genders (p>0.05). Nevertheless, a significant difference was observed between July 2013 (P3) and March 2014 (P5) among children aged 3–6 years (p<0.05); between these same children and also adults [≥19 years] in October 2013 (P4) (p<0.05), or the group of over five years older children [7–10 years] also in 2013 (P4) and [15–18 years] in June 2013 (P2) (p = 0.04) (Fig 2).

The prevalence of ME + was significantly higher in individuals of 3–14 years) and whom of 15–18 years than in adults of 19 years old and more (p<0.05) (Fig 2). The PCR+ prevalence among ME—participants did not differ significantly across the age groups. The overall prevalence of *P*. *falciparum* infection was significantly higher in individuals less than 18 years old than in older individuals (respectively 32.7% and 9.6%, p<0.001) (Fig 2).

**Fig 3:**
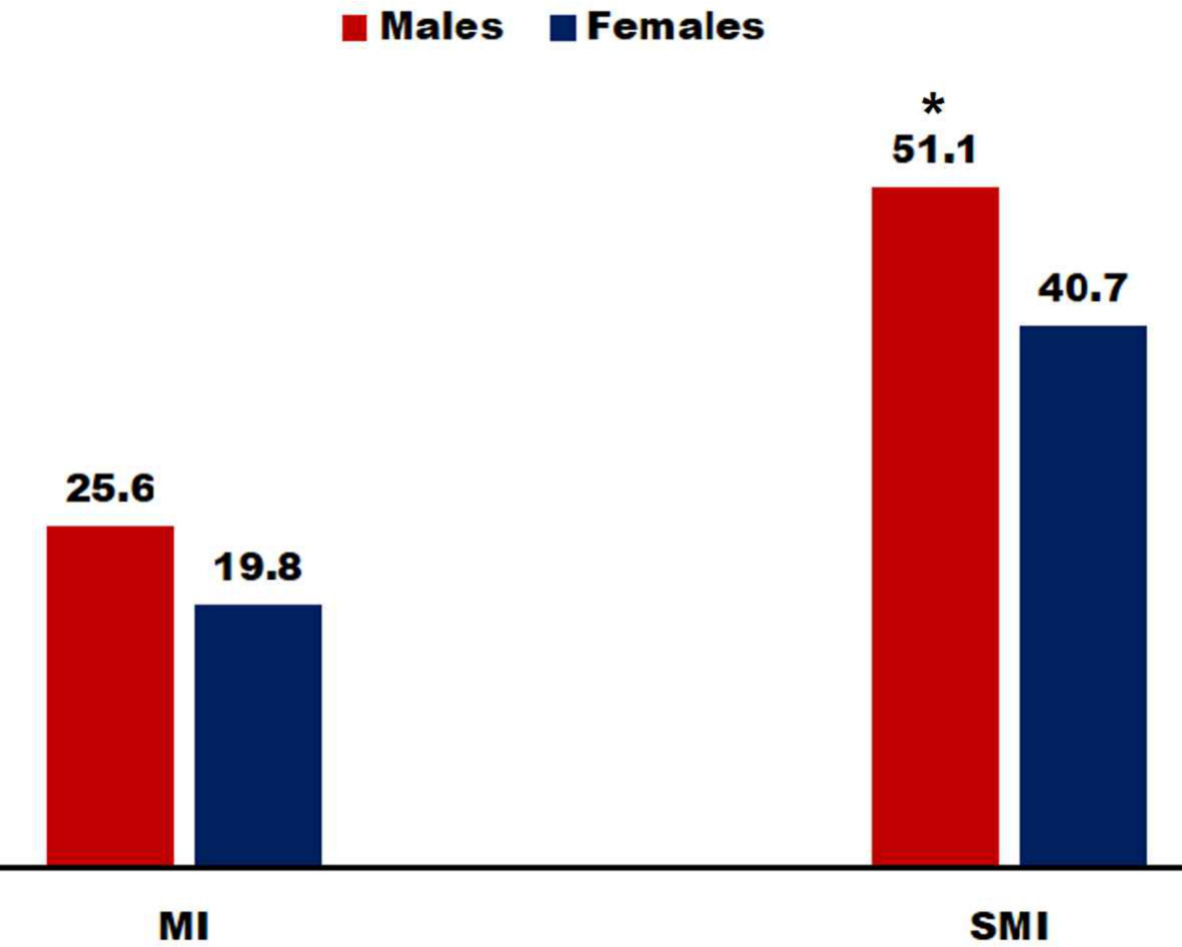
Mean prevalence according to the age groups. * Significant difference between the prevalence of males obtained by PCR and that observed among females.

The prevalence of both ME+ and PCR+ was slightly but not significantly higher in males than in females (Fig 3).

### Parasite parameters

Although peripheral blood parasites were almost all trophozoites (50 to 150 000 parasite per μl), several gametocytes were identified in 35 ME + individuals (9 symptomatic and 26 asymptomatic) during all the periods except P6, with 11 (11.8%) in P1; 5 (6.9%) in P2; 8 (14.0%) in P3; 5 (13.9%) in P4; and 3 (5.9%) in P5). Gametocyte density ranged from 50 to 700 per μl.

### Malaria and fever

Thirty febrile individuals were identified: 9/370, 2.43% in P1; 1/267, 0.37% in P2; 4/218, 1.83% in P3; 1/255, 0.39% in P4; 14/232, 6.03% in P5; and 1/145, 0.68% in P6, with a mean of 1.95%. *P*. *falciparum* was detected in 23 of these 30 febrile subjects (76.7%; 17 ME + and 6 PCR+). Eight of the 17 ME + patients had at least 5000 parasites per μl (S1 Annexe). Thus, only 8 patients were considered to have clinical malaria, defined by fever associated with at least 5000 trophozoites per μl of blood.

### Attitudes and behaviors concerning malaria

ITNs were used by 33.47% of individuals (95%CI [27.64–38.73]) and IRS by 18.26% (95%CI [13.8–23.8]). No significant difference was observed in the use of ITNs between *P*. *falciparum*-infected and uninfected individuals. However, adults used ITNs more than individuals ≤16years old. Self-medication was declared by 12.22% (95%CI [8.55–17.18]) of individuals, with no significant difference between clinical groups (infected and uninfected), genders or age groups (Table 4).

**Table 4:** Attitudes and behaviors concerning malaria.

	ITNs	P value	IRS	P value	Self-medication	P value
**Population follow up in March 2014 (N = 232)**	76 (32.76)		41 (17.67)		28 (12.00)	
**Positive malaria infection (N = 134)**	37 (28.46)	0.072*	26 (20.00)	0.361*	18 (13.85)	0.293*
**Negative malaria infection (N = 98)**	39 (39.80)		15 (15.31)		9 (9.28)	
**Males (N = 115)**	30 (26.08)	0.929**	21 (18.26)	0.662**	14 (12.17)	0.813**
**Females (N = 117)**	46 (39.32)		20 (17.09)		14 (11;97)	
**≤15 (N = 159)**	38 (26.57		28 (19.58)		17 (11.89)	
**≥16 (N = 73)**	38 (44.71)	0.003***	13 (15.29)	0.470***	10 (11.90)	0.804***

**N:** frequency

**ITNs:** Insecticide Treatment Nuts

**IRS:** Indoor Residual Spread

Data are presented as effectives and frequencies in brackets. The symbol * is the p value traducing the comparison between the positive infection malaria and negative in each rubric (INTs, IRS and self-medication). The second symbol ** is the comparison between Males and females. The last symbol *** compares the use of the INTs, IRS and the practice of self-medication between people less than 16 years and those high than 15 years

In logistic regression analysis, even if the associations are little ITNs, IRS and self-medication were associated with protection from *P*. *falciparum* infection, respective odds ratios were 0.60 (0.34–1.05), 1.38 (0.69–2.75) and 1.57 (0.68–3.59). When we analyzed according to age, protection against *P*. *falciparum* infection was associated with the use of ITNs: adults tended to be more protected than younger individuals, with an odds ratio of 2.43 (1.36–4.34) (Table 5).

**Table 5:** Stepwise multivariate logistic regression analysis of significant variables.

	ITNs	IRS	Self-medication
	OR (95%CI)	OR (95%CI)	OR (95%CI)
N = 232	n = 76	n = 41	n = 28
**Malaria infection**			
Positive (n = 134)	1,00	1,00	1,00
Negative (n = 98)	0.60 (0.34–1.05)	1.38 (0.69–2.75)	1.57 (0.68–3.59)
**Gender**			
Females (n = 117)	1,00	1,00	1,00
Males (n = 115)	0.97 (0.47–2.00)	0. 81 (0.32–2.05)	0.83 (0.19–3.48)
**Age (years)**			
≤18 (n = 159)	1,00	1,00	1,00
≥19 (n = 73)	2.43 (1.36–4.34)	0. 76 (0.36–1.60)	0.90 (0.38–2.11)

## Discussion

The decline in the prevalence of malaria observed in many countries, including Gabon, has been attributed to artemisinin-based combination therapy (ACT), monitoring of pregnancies, and distribution of insecticide-treated nets. For example, in 2008 the proportion of *Plasmodium*-positive slides was reported to have fallen considerably in Libreville, the capital of Gabon [27], as well as in Franceville in the southeast [19,28]. However, these studies involved symptomatic patients usually recruited from pediatric units. In addition, there are few data on rural areas.

This longitudinal study revisited the situation in Dienga 10 years later, focusing on the hidden reservoir of *P*. *falciparum* infection. The study was designed to determine the true prevalence of *P*. *falciparum* infection during six passages (P) involving 370 individuals. The overall *P*. *falciparum* carriage rate (ME + and PCR+) ranged from 57.7% in P5 to 34.1% in P4, with an average of 45.9% (23.2% ME + and 22.7% PCR+). This study provides one of the most accurate field estimates of *P*. *falciparum* prevalence across all age groups in Gabon. Indeed, previous studies focused only on children [19,21,29] or pregnant women [30,31]. A high level of *falciparum* infection among asymptomatic individuals was found in Dienga, confirming the results of previous studies conducted in the same location [20], as well as in Lambaréné, central Gabon [32], and Kenya [33]. The prevalence of microscopic *P*. *falciparum* infection in Dienga (23.2%) was very similar to that found in 2011 among febrile children in Libreville (24.1%), an urban area of northwest Gabon [34]. Assuming that asymptomatic *P*. *falciparum* carriers have a five-fold increase in the risk of developing malaria [35], such carriers should be taken into account in epidemiological surveillance, notably in rural areas.

No significant difference was observed in the overall prevalence of *P*. *falciparum* across our six survey periods. In 2001, Elissa et al. reported that malaria transmission was perennial in Dienga, with a major peak in December to March and a minor peak in July to August; malaria transmission was almost undetectable during the rest of the year, owing to the disappearance of the main vector, *Anopheles gambiae sensu lato*, in the dry season [23,36]. It is conceivable that climate change (notably rainfall), together with other environmental and behavioral changes, influenced the abundance of *A*. *gambiae s*.*l*. during the 10-year period in question. Entomological investigations will be necessary to confirm this hypothesis, as well as the possible introduction of other vectors.

The mean overall prevalence of *P*. *falciparum* infection was similar in children (3–6, 7–10 and 11–14 years) and adolescents (15–18 years) but higher than in adults (Fig 3), as was the microscopic prevalence (Fig 2). Together, these data suggest that adolescents are being infected by *P*. *falciparum* as frequently as younger children, suggesting a shift in the age group at risk and therefore a change in malaria epidemiology in this region. Similar results have been obtained both in field studies [37,38] and pediatric units [21]. Children under 5 years of age are the most vulnerable group and are commonly targeted by intervention strategies, possibly explaining the higher relative prevalence in older children and adolescents. Both immunity and the attitudes of adolescents can also influence the risk of developing malaria [39]. *P*. *falciparum* carriers were slightly but not significantly more frequent among males than females (Fig 3).

Only 30 febrile individuals were identified, 8 of whom appeared to have clinical malaria, defined as fever associated with at least 5000 trophozoites per μl of blood [23]. The remaining 15 individuals had low parasitaemia, including SMI, and may have had other infections. Nevertheless, SMI (ME—PCR+) can be associated with severe malaria in areas of both discontinuous [6] and perennial transmission [40]. Submicroscopic infection has also been linked to maternal anemia and low birth weight [41,42]. The prevalence rates of microscopic and submicroscopic *P*. *falciparum* infection were both comparable, confirming that almost 50% of carriers maybe missed by microscopy alone [5].

Gametocytes were identified in 32 symptomatic or asymptomatic microscopy-positive individuals. As we did not seek to detect submicroscopic gametocyte carriage, the true prevalence of gametocyte carriage may have been underestimated. In the absence of treatment, individuals carrying asexual parasites could potentially produce sexual gametocytes that can infect mosquitoes [43]. The *P*. *falciparum*-infected adults and children identified in this study likely represent a parasite reservoir that may sustain malaria transmission [44]. Submicroscopic infection, including gametocyte carriage, must thus be taken into account in malaria control programs [5,44].

In February 2003, a prospective cohort of 319 subjects 13–70 years old was followed for malaria and hepatitis virus co-infection during a one-year period in Dienga. The overall prevalence of malaria parasitemia was 20.8% (7.2% ME + and 13.6% PCR+) [20,45]. This microscopic prevalence of 7.2% was close to the pre-elimination threshold of 5%. In the present study, 10 years later, we found by microscope a significant higher prevalence of 23% (p < 0.001). The overall prevalence of *P*. *falciparum* infection was also significantly higher in 2013 than in 2003 (45.9% versus 20.8%) (p<0.05). This increase could be due to several factors. First, in 1994 CIRMF established a fully equipped field base with three permanent workers (one nurse and two technicians) ensuring full-time cohort monitoring. In addition, the base was supplied monthly with food and drugs and was visited by a CIRMF physician. All febrile patients in Dienga were examined, and those with malaria were treated free of charge. The antimalarials used at the time were mainly chloroquine and sulfadoxine pyrimethamine (SP). In 2006, CIRMF research activities in Dienga were reduced, and in 2008 they were shifted to other topics. Antimalarial drugs were no longer distributed to the population and patient management was provided only by the local health center in Dienga, that had limited resources. Following the change in the national antimalarial policy, with a switch to ACT, in 2005 the Gabonese Malaria National Control Program (MNCP) launched a nationwide campaign of ITNs distribution, intermittent preventive treatment (IPT), and educational messages. These interventions have been evaluated in many parts of the country [27,46,47] but not in Dienga. A significant decline in the malaria burden led to a deceleration of preventive activities in 2008 [27].

The study found that ITNs and IRS were used by respectively 33.47% and 18.26% of respondents, and self-medication by 12.22%. In 2003, the rate of self-treatment was 11.3% in Dienga [45].

The comparison in the use of ITNs between youngers (3–15 years old) and individuals of 16 years old and more has shown a significant difference (p = 0.003) (Table 4), the adult individuals were using more ITNs and they were less infected. Perhaps these results can be explained by the attitude of adolescents and the absence of children less than 3 years old in the study population. Logistic regression analysis showed that the use of ITNs was a protective factor against *P*. *falciparum* malaria in Dienga (Table 5). However, the fact that the adults were less frequently infected was not necessarily due to the use of ITNs. The oral survey of ITNs and IRS use was performed in March 2014, corresponding to the major peak of malaria transmission. Of interest, the highest prevalence of *P*. *falciparum* malaria (57.7%) was observed in March 2014 (Fig 1). ITNs coverage was lower than the national average of 57% reported in 2008 [48] and this could explain the apparent lack of protection.

These results show that the MNCP campaign has not yielded the hoped-for results. The increase in malaria prevalence among febrile children observed in several pediatric units has been attributed to the decline in the preventive activities of the MNCP [21]. This may also explain the present results; although the study was performed on a cohort of asymptomatic individuals. A rebound of malaria has also been observed in Kenya [49]. In contrast, no such increase has been reported in countries where interventions have been sustained [50,51]. Finally, factors such as mosquito resistance to conventional insecticides could further undermine preventive measures. Such resistance has been documented in Libreville and Port-Gentil [52].

It would have been interesting to perform, as planned, passages every two months for a full year. Unfortunately, because of logistic constraints, it was not possible to achieve this goal. Consequently, the six passages were carried out irregularly during a 15-month period.

In conclusion, the study showed a near-threefold increase in the prevalence of *P*. *falciparum* infection in Dienga, southeastern Gabon, over a recent 10-year period. Although a large proportion of infected subjects were asymptomatic, the risk of older children and adolescents of developing *falciparum* infection becomes higher. These results reveal an urgent need for reinforcing intervention strategies.

## Supporting Information

S1 Annexe Febrile individuals over the six passages of follow up. (DOC)

S1 Questionnaire Questionnaire and informed consent. (DOC)
