# Supplementary material for: Marked Rise in the Prevalence of Asymptomatic Plasmodium falciparum Infection in Rural Gabon
Source: PLoS One. 2016 May 26;11(5):e0153899. doi: 10.1371/journal.pone.0153899 (PMC4881998; doi:10.1371/journal.pone.0153899)
Supplement: S1 Annexe — (DOC) [file pone.0153899.s001.doc]

**Annexe1**: Febrile individuals over the six passages of follow up

| **Sample name** | **Age (year)** | **gender** | **Parasite density (parasite/µl)** | | | | | |
| --- | --- | --- | --- | --- | --- | --- | --- | --- |
|  |  |  | April 2013 | June 2013 | July 2013 | October 2013 | March 2014 | June 2014 |
| **ME 23** | 9 | M | 30,125 |  |  |  |  |  |
| **ME 24** | 15 | M |  |  |  |  | 250 |  |
| **ME 29** | 11 | M |  |  |  |  | 50 |  |
| **ME 53** | 10 | M | 3,950 |  | 100 |  |  |  |
| **ME 73** | 13 | M | 5,000 |  |  |  |  |  |
| **ME 82** | 5 | M | 50 |  |  |  | 4,050 |  |
| **ME 85** | 4 | F |  |  |  |  | 100 |  |
| **ME 86** | 16 | F |  |  |  |  | 250 |  |
| **ME 90** | 6 | F |  |  |  |  | 2,150 |  |
| **ME 101** | 6 | M | 150, 000 |  |  |  |  |  |
| **ME 124** | 8 | F |  |  | 52,650 |  |  |  |
| **ME 128** | 69 | M |  |  |  |  | 650 |  |
| **ME 139** | 9 | F |  |  | 100 |  |  |  |
| **ME 148** | 14 | M |  |  |  | 350 |  |  |
| **ME 153** | 14 | M |  |  |  |  | 50 |  |
| **ME 154** | 14 | M |  |  |  |  | 50 |  |
| **ME 191** | 7 | F | 2,000 |  |  |  |  |  |
| **ME 193** | 8 | M | 2,500 |  |  |  |  |  |
| **ME 210** | 7 | F |  | 25,150 |  |  |  |  |
| **ME 217** | 9 | F |  |  |  |  | 550 |  |
| **ME 227** | 8 | M | 18,150 |  |  |  |  |  |
| **ME 231** | 8 | M |  |  |  |  | 1,750 |  |
| **ME 242** | 10 | F |  |  |  |  | 500 |  |
| **ME 274** | 4 | M | 5,850 |  |  |  |  |  |
| **ME 286** | 38 | F |  |  | 400 |  |  |  |
| **ME 340** | 84 | M |  |  |  |  |  | 25,333 |
| **ME 378** | 4 | M |  |  |  |  | 2,050 |  |
| **ME 382** | 29 | F |  |  |  |  | 100 |  |
| **Total :** | | | 9 | 1 | 4 | 1 | 14 | 1 =**30infections** |

The individual ME 82 had a malaria infection in April 2013 and in March 2014; the ME 53 had an infection in April and another in July in the same year (2013).

*ME: ID*

*M: male*

*F: female*
