# Supplementary material for: Marked Rise in the Prevalence of Asymptomatic Plasmodium falciparum Infection in Rural Gabon
Source: PLoS One. 2016 May 26;11(5):e0153899. doi: 10.1371/journal.pone.0153899 (PMC4881998; doi:10.1371/journal.pone.0153899)
Supplement: S1 Questionnaire — (DOC) [file pone.0153899.s002.doc]

**CENTRE INTERNATIONAL**

**DE RECHERCHES MEDICALES**

**DE FRANCEVILLE**

**BP 769 Franceville Gabon**

**Tel : (241) 67 70 92/ 67 70 96**

**Fax : (241) 67 72 95/67 79 30**


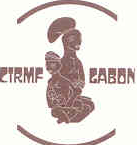


**Questionnaire and informed consent**

**MALARIA MOLECULAR EPIDEMIOLOGIE In DIENGA**

1. **Identification Number: ……………………… N° in Laboratory: ……………………………….**
2. **Name: ……………………………………………………………………………………**
3. **Year of Born : …………………………………………………….….../…/…/………/…/…/……/…/…/…/…/**

*(Day) (Month) (Year)*

1. **Sex :……………………………………………………..……… □ Male^1^ □ Female^2^ [__]**
2. **Street in the village: ……………………………………………………………………………………….**

**Study Goals :**

In Gabon, most studies that describe malaria epidemiology are confined in urban and hospital settings. This situation leads to poor information about rural areas and asymptomatic infections.

In 2013, malaria caused 584 000 (367 000–755 000) Deaths around the world; 78% of which in sub-Saharan Africa . Manny Children, who survive to cerebral malaria, may have troubles in learn or brain dysfunctions

The aim of this is to determine the true prevalence of *P. falciparum* infection in Dienga.

**Procedures**

After obtaining your informed consent, the CIRMF physician will examine you and a sample of your blood will be taken for the laboratories tests. The sampling procedure is sometime difficult but our team takes his charge. All result will be confidential and you will be treated if necessary.

**Potential benefits of the study**

All participants in this study will benefit of a checkup (Malaria, Hematology, HIV, Syphilis, Hepatics B and C, Fillariose, free of charge and only taken by charge by the CIRMF laboratory.

By being included, you are free and if you want go out of this study, you never will be constrained. You can refuse at all the moment to be picked up.

**Investigator**

**Participant, parent or legal guardian signature**

**Tel.**

**Date :**

**Signature :**

**FICHE PATIENT**

**PATIENT IDENTIFICATION:**

1. **Inclusion date …………………………………….....…/…/…/………/…/…/……/…/…/…/…/**

*(Day) (Month) (Year)*

1. **Site  : …………………………………………….** [__]
2. **Numero of sample: ………………………. N° of patient:……...…………………………….….**
3. Do you sleep in Insecticide Treat bed net? □ No^0^ □ Non-treated^1^ □ Treated^2^ [__]
4. Did you have a fever in the last week? ………Day
5. Did you take an antimalarial? (DCI) ……………………………………………………… [__]

**CLINICAL EXAMINATION**

1. Temperature: [__] [__], [__] °C…... [ ] axillar …………….or…………… [ ] rectal
2. Deshydratation? □ Yes^1^ □ No^0^ [__]
3. Nutritional state? □ Good^1^ □ Bad^2^ [__]
4. Shaking / Vomiting : □ Yes^1^ □ No^0^ [__]
5. Abdominal Dolores : □ Yes^1^ □ No^0^ [__]
6. Constipation / Diarrhea □ Yes^1^ □ No^0^ [__]
7. Icterus : □ Yes^1^ □ No^0^ [__]
8. Splenomegaly : □ Yes^1^ □ No^0^ [__]
9. Cough or respiratory difficult: □ Yes^1^ □ No^0^ [__]
10. ORL Path (Angina, laryngitis, pharyngitis, otitis) □ Yes^1^ □ No^0^ [__]
11. Head ach : □ Yes^1^ □ No^0^ [__]
12. Prostration – Confusion : □ Yes^1^ □ No^0^ [__]
13. Blantyre Score:

**Motor** **Verbal Eyes mouvements**

[ ] Normal response : 2 [ ] Normal dialogue : 2 [ ] Well: 1

[ ] Late response: 1 [ ] Anormal dialogue : 1 [ ] Bad: 0

[ ] No response: 0 [ ] No response : 0

**Total of Blantyre score** [___]/5 [__]

**BIOLOGICAL CRITERIAS**

1. Plasmodiale species : □ *falciparum*^1^ □ *malariae*^2^ □ *ovale*^4^ □ *vivax*^5^ [__]
2. Parasite : □ Trophozoïte^1^ □ Schizontes^2^ □ Gametocytes^4^ [__]
3. Parasitemia :………………..…**HP/µl** □ <1 000^1^ □ Entre 1 000 à 5 000^2^ □ > 5 000^3^ [__]
4. White blood cells rate  :……………………x1 000 **/** **mm^3^**
5. Red blood cells rate : ……………………x1 000 **/ mm^3^**
6. Plaquets rate:……………………….. x1 000 **/ mm^3^**
7. Hemoglobin (Hb) :………………………… **g/dl**
8. Hematocrits:………………………………… **%**
9. VGM :……………………………………….. **Fl**
10. TCMH :……………………………………… **pg**
11. CCMH : …………………………………….. **g/dl**
12. Glycaemia  : ……….………………. **mmol/l**
13. Hemoglobin Electrophoresis □ AA^1^ □ AS^2^ □ AS^3^ [__]
14. Rhesus and Blood Group: ………………………

**DIAGNOSTIC AND CONCLUSION**

1. Asymptomatic
2. Acute malaria: □ Simple^1^. □ Moderate^2^ (Non per os) □Severe^3^ [__]
3. Anemia: □ No^1^. □ Moderate^2^ □ Severe^3^ [__]
